# Developing patient safety scale for hospitals

**DOI:** 10.55730/1300-0144.5810

**Published:** 2024-01-05

**Authors:** Metin DİNÇER, Esra KARATAŞ OKYAY, Yunus Emre KARATAŞ, Erol GÖRAL

**Affiliations:** 1 Health Management Department, Faculty of Health Sciences, Ankara Yıldırım Beyazıt University, Ankara, Turkiye; 2 Department of Midwifery, Faculty of Health Sciences, Kahramanmaraş Sütçü İmam University, Kahramanmaraş, Turkiye; 3 Doctorate Program in Health Management, Social Science Institute, Hacettepe University, Ankara, Turkiye; 4 Master Program in Health Management, Health Science Institute, Ankara Yıldırım Beyazıt University, Ankara, Turkiye

**Keywords:** Patient safety, culture, scale, validity, reliability

## Abstract

**Background/aim:**

The study aimed to contribute to the literature with a reliable and valid scale for hospitals to be used in determining the current patient safety culture and following up on its development.

**Materials and methods:**

The study was conducted with the participation of 1137 healthcare professionals selected using the convenience sampling method in 3 secondary-care state hospitals and three research and training hospitals, one of which was affiliated with a medical faculty, and two were affiliated with the Health Sciences University. To begin with, to discover the latent structure of the items on the scale, an Exploratory Factor Analysis (EFA) was performed. Additionally, to determine the factor structure of the scale, the Confirmatory Factor Analysis (CFA) method was used. The Cronbach’s alpha coefficient was calculated to check the reliability of the responses.

**Results:**

According to Kaiser-Meyer-Olkin (KMO = 0.924) coefficient and the result of Bartlett’s test of sphericity (*χ*^2^ = 9748.777, df = 770), it was determined that the data structure was suitable for factor analysis. The Cronbach’s alpha coefficient of the total scale was found to be 0.921. According to the EFA results, the scale was determined to have seven subscales, which were 1. Organizational Learning, Development, and Communication, 2. Management Support and Leadership, 3. Reporting Patient Safety Events, 4. Number of Personnel and Working Hours, 5. Response to Error, 6. Teamwork, and 7. Working Environment. The goodness-of-fit index results of the scale showed a good model fit (*χ ^2^*/ df = 3.04, RMSEA = 0.06, CFI = 0.97, NFI = 0.95, IFI = 0.97, SRMR = 0.06). The Cronbach’s alpha coefficients of the subscales varied between 0.66 and 0.91.

**Conclusion:**

The results showed that the Patient Safety Scale for Hospitals is a valid and reliable measurement instrument for healthcare professionals.

## 1. Introduction

Unsafe healthcare practices cause 3 million deaths worldwide per year, as well as costing $606 billion in developed countries [1]. In the United States, patient safety violations are reported to increase the length of hospital stay by an estimated 2.4 million days, with an additional cost of $9.3 billion (£7.3 billion; €8.2 billion) [2]. It is estimated that 43.5% of patient safety-related adverse events occurring in the hospital could have been prevented [3]. In hospitals in low- and middle-income countries, 134 million adverse events occur each year, resulting in over 2.5 million lost lives [4]. While 10% of patients in developed countries are likely to be harmed, this rate is estimated to be 18% in African and Eastern Mediterranean countries [5].

Strategies for patient safety based on the principle of protecting patients from preventable harm, i.e. first do no harm [6], are a collective product of individual work and teamwork determined by the attitudes, perceptions, competencies, and behavioral patterns of employees [7]. It is, therefore, necessary to create a patient safety culture that identifies potential errors that could harm patients, consistently reduces the occurrence of errors, and learns from these errors [8]. The most important factors in terms of a patient safety culture are management support and leadership because, without these, a patient safety culture cannot be created [9]. Other topics that will contribute positively to a patient safety culture and that top management should lead are encouraging teamwork, creating working conditions to reduce errors, improving organizational development and communication, not treating errors with a punitive approach, and establishing an appropriate error reporting system [10–16].

In this study, it was aimed to develop a new scale because patient safety remains an essential public health problem [17], developing countries are more prone to patient safety violations [5], and the view of patient safety is different in developed countries due to the fact that patient safety practices have started earlier [18], there is a need for the development and learning of a safety culture in organizations depending on individual, group, and organizational factors and time for this [19], and there is a need for a scale developed within an existing organizational culture to measure the patient safety culture of healthcare professionals as a team. It is seen that the patient safety scales that are currently being used in Türkiye are adapted [20, 21] or focused on specific healthcare professionals such as nurses [22]. For these reasons, there is a need to develop a new instrument to measure the approach of healthcare professionals, to a patient safety culture as a team. Additionally, the scale developed in this study can be used by healthcare organizations that are currently working on the creation of a patient safety culture.

## 2. Materials and methods

### 2.1. Ethical aspects of the study

Ethical approval for the study was obtained from Yıldırım Beyazıt University Health Sciences Ethics Board, with the decision dated 07.04. 2022 and numbered 06. The general permission for the institutions where the study would conduct was received from the General Directorate of Health of Public Hospitals under the Turkish Ministry of Health with the decision dated 07.07.2022 and numbered E-32693113-622.03-03-552. This permission was then sent to institutions in the province of Ankara by the letter of the Ankara Provincial Directorate of Directorate dated 06.09.2022 and numbered E-90739940-799-1921. Finally, written permissions for the study were obtained from the hospitals to be included in the study.

The hospitals to be included in the study were selected using the convenience sampling method. Interviews were held with the managers of hospitals, and those who agreed for their institutions to be included were included in the study. The three secondary-care state hospitals which were included in the study have bed capacities of 100, 106, and 250. Of the three research and training hospitals included in the study, the two affiliated with the Health Sciences University have bed capacities of 418 and 760, and the one affiliated with the Medical Faculty of a university in Ankara has a capacity of 264 beds. Healthcare professionals who would participate in the study were selected using the convenience sampling method. The draft scales used in the study were printed on paper and delivered to the management teams of the hospitals, who distributed them to their employees who agreed to participate, collected them back after the participants filled them out, and delivered them to the researchers.

### 2.2. Data collection instrument

The scales used in the literature and items on these scales were examined. Additionally, the “Quality Standards in Health for Hospitals” issued by the General Directorate of Healthcare Services [23] and the “Standards of Accreditation in Health Hospital Kit” [24] issued by the “Türkiye Healthcare Quality and Accreditation Institute” were analyzed. A 73-item draft item pool under ten subscales was created for the draft Patient Safety Scale for Hospitals. The subscales and the numbers of items under these subscales were: 1-Teamwork, seven items; 2-Working Conditions, twelve items; 3-Organizational Development, five items; 4-Response to Error, four items; 5-Upper Management Support, ten items; 6-Error Reporting, seven items, 7-Communication, twelve items, 8-Patient Transfer and Shift Change, three items, 9-Job Satisfaction, eight items, and 10-Perceived Stress, five items. The draft scale was created as a 5-point Likert-type scale, and the response options of each item were “1- Strongly Disagree”, “2- Disagree”, “3- Undecided”, “4- Agree”, and “5- Strongly Agree.”

The draft scale was reviewed by 12 field experts. While reviewing, the experts were asked to choose one of the four options: “Not Suitable”, “Partially Suitable”, “Quite Suitable”, and “Very Suitable.” If they chose “Not Suitable” or “Partially Suitable”, they were asked to justify their decision to do so. The draft scale was also evaluated by a Turkish language expert. In the next phase, the draft scale was applied in a pilot implementation to 35 healthcare professionals working in a tertiary hospital and a secondary state hospital. Following the pilot implementation, necessary adjustments were made to the draft scale, and considering the number of items on the scale and the expected required time for the respondent to fill out the scale, nine subscales were included. These subscales were: 1-Teamwork, seven items; 2-Personnel, five items; 3-Organizational Learning, four items; 4-Response to Error, six items; 6-Error Reporting, four items; 6-Communication Related to Error, four items; 7-Upper Management Support and Leadership, eight items; 8-Important Patient Information, three items, and 9-Working Environment, three items. The revised draft scale was assessed by a Turkish language expert, and expert opinions were taken into consideration. The study was conducted with this final form of the draft scale.

### 2.3. Limitations

It is assumed that the answers given by the participants in the study were sincere and accurate. The participants of the study were limited to physicians, nurses, midwives, emergency medical technicians, anesthesia technicians, laboratory technicians, and radiology technicians working in the included hospitals.

### 2.4. Data analysis

For testing the construct validity of the scale, the exploratory factor analysis (EFA) and confirmatory factor analysis (CFA) methods were used. EFA is a multivariate statistical method that aims to determine multiple interrelated variables and fewer theoretically significant latent variables [25]. It is a type of validity test used in determining the number of factors in scale development studies. The draft scale was filled out by 1180 healthcare professionals, missing data were checked, the data of the participants who did not respond to all items on the scale and those who gave the same response to all items were excluded from the analyses, and 1137 participants in total completely responded to the scale. Among the healthcare professionals who participated in the study, 13.28% (n = 151) were physicians, 74.76% (n = 850) were nurses, midwives, or emergency medical technicians, and 11.96% (n = 136) were anesthesia, laboratory, or radiology technicians.

The entire dataset was randomly divided into two datasets according to the total number of participants, and EFA was applied to one of these two datasets. Through EFA, the final factor structure was obtained, and confirmation was aimed at applying CFA to the other dataset. Researchers frequently use the method of dividing the data in half completely at random. It was reported that randomly splitting the dataset in half in large samples did not create a difference in the results [26]. In the data analyses in this study, EFA was performed using the IBM SPSS 22 software, while the LISREL 8.80 software was employed for CFA. Reliability was tested using the Cronbach’s alpha coefficient.

#### 2.4.1. Exploratory factor analysis

EFA is a factor reduction method, and there are various views regarding the identification of the number of factors. Having an eigenvalue greater than 1, which is known as Kaiser’s K1 rule, is the essential method for deciding on the number of factors [27]. The Kaiser-Meyer-Olkin (KMO) test and Bartlett’s test of sphericity were performed to determine whether the data were suitable for factor analysis [28]. There are also various criteria regarding the factor load values required for items on loaded on factors. According to a previous study, for an item to be related to a factor, the minimum factor load value must be 0.30 [28]. Additionally, when an item is loaded on multiple factors, the difference between the factor loads of the same item must be at least 0.10 [28].

The maximum probability method, which is recommended to be used in large samples as a factor extraction method, and the direct Oblimin method, which is a rotation method, were used in this study. It was previously stated that there are no significant differences between factor extraction methods used in EFA in terms of the number of factors and that methods may differ only if there are low factor load values [27].

An EFA was applied to the data obtained from 578 participants. Items 2, 3, 8, 11, 13, 14, 15, 22, 24, 34, 36, and 42 were inversely scored items, and they were included in the analyses accordingly.

#### 2.4.2. Confirmatory factor analysis

To verify the 39-item and 7-subscale construct obtained after conducting the EFA, a CFA was applied to the second dataset. CFA is a multivariate statistical method that verifies a theorical construct related to a model with a known factor structure [30]. For the CFA to be conducted in this study, new numbers were assigned to the items. The codes of the items were changed to Organizational Learning Development and Communication (OLDC) for subscale 1, Management Support and Leadership (MSL) for subscale 2, Reporting Patient Safety Events (RPSE) for subscale 3, Personnel and Working Hours (NPWH) for subscale 4, Response to Error (RE) for subscale 5, Teamwork (TW) for subscale 6, and Working Environment (WE) for subscale 7. t-values for all scale items being at the significance level of 0.05 and being outside the ±1.96 interval, which is the t-value at the infinite degrees of freedom, show that the factor load value is significant [25]. As the absolute t-values of all items on the scale were greater than 1.96, they were statistically significant, and there were no items that should have been removed from the scale.

The chi-squared value tends to be significant when large samples are used [31]. Therefore, the ratio of the chi-squared value to the degrees of freedom value is recommended for use [32]. A ratio lower than three shows an excellent fit, while a ratio between 3 and 5 shows a good fit [30]. On the other hand, in general, there is no consensus on what a good level of fit is. The aforementioned ratio could be lower than three and can reach up to 5 [33–35]. Another goodness-of-fit index that is used in this method is RMSEA (Root Mean Square Error of Approximation). An RMSEA value lower than 0.08 shows a good fit, while one lower than 0.05 indicates an excellent fit [36]. Other model fit indices that were used in this study were CFI (Comparative Fit Index), NFI (Normed Fit Index), IFI (Incremental Fit Index) [37], and SRMR (Standardized Root Mean Square Residual). A more detailed explanation of these fit indices was reported by Schumacher and Lomax [36].

## 3. Results

### 3.1. Exploratory factor analysis results

First, the Kaiser-Meyer-Olkin (KMO) (0.924) and Bartlett’s test (*χ*^2^ = 9748.777, df = 770, p = 0.000) results were analyzed. Accordingly, it was determined that the data structure and sample were suitable for factor analysis. According to the results obtained by administering the 39-item Patient Safety Scale for Hospitals, eigenvalues were higher than one for seven subscales. All subscales collectively explained 49.2% of the total variance in the measured variable.

The factor loads values of items were found to be between 0.325 and 0.674 for OLDC, between −0.780 and −0.402 for MSL, between 0.509 and 0.761 for RPSE, between 0.311 and 0.739 for NPWH, between 0.342 and 0.423 for RE, between −0.822 and −0.386 TW, and between 0.398 and 0.650 for WE. Therefore, factor loads in all structures were significant. Initially, the draft scale had 44 items. Items 15, 16, 29, and 34 did not yield any significant load on any factor, item 40 was removed from the scale by the researchers, and the EFA results for the 39-item scale are presented in Table 1 as items factor loadings of items and in Table 2 as subscales’ values of eigenvalue, variance explained and Cronbach’s alpha.

### 3.2. Confirmatory factor analysis results

The t-values of each item that were obtained as a result of the CFA are presented in Table 3. The graph of the standardized path coefficients for the Patient Safety Scale for Hospitals is shown in Figure. The path coefficients were found to be in the ranges of 0.45–0.76 for the OLDC, 0.58–0.80 for the MSL, 0.45–0.81 for the RPSE, 0.31–0.71 for the NPWH, 0.43–0.78 for the RE, 0.69–0.82 for the TW and 0.53–0.67 for the WE. These coefficients were found to be high.

The model data fit results are presented in Table 4. Among the goodness-of-fit indices that were used in the study, The RMSEA value was found as 0.060, showing a good fit. Considering the other goodness-of-fit index values, for the Patient Safety Scale for Hospitals, model data fit was achieved (RMSEA = 0.060, CFI = 0.97, NFI = 0.95, IFI = 0.97, SRMR = 0.060). The ratio of chi-squared-to-degrees of freedom ratio was found as 3.04.

### 3.3. Reliability

The Cronbach’s internal consistency coefficient of the total scale was found as 0.921. The results of the reliability analysis of the subscales obtained in the study are presented in Table 3. The coefficients of the seven original subscales were as follows: OLDC = 0.91; MSL = 0.90; TW = 0.85; WE = 0.71; RPSE = 0.66; NPWH = 0.66; and RE = 0.66. As subscales, OLDC had thirteen items, MSL had eight items, TW had four items, and these subscales were determined to be highly reliable. Moreover, WE had four items, RPSE had three items, NPWH had four items, and RE had three items, and these subscales were determined to be quite reliable. However, it is also known that it is possible to obtain a low Cronbach’s alpha coefficient when the number of items is low.

## 4. Discussion

The KMO coefficient provides information about the suitability of both the data matrix for factor analysis and the data structure for factor extraction, while Bartlett’s test can be considered evidence of both the suitability of the data matrix and the normality of the scores [28]. In this study, according to the result of the KMO test, the data were suitable for factor analysis, and based on the result of the Bartlett’s test of sphericity, the data were determined to be normally distributed for factor analysis [35, 38]. Some KMO and Bartlett’s test results reported in various cultural adaptations of scales are as follows: the Hospital Survey on Patient Safety Culture in Palestine (HSOPSC-AR) KMO = 0.85, Bartlett’s test, p < 0.001 [39]; the Hospital Survey on Patient Safety Culture in Türkiye (HSOPS-TR) KMO = 0.9, Bartlett’s test, p < 0.001 [21]; the Hospital Survey on Patient Safety Culture in China (HSPSC-CN) Version KMO > 0.7, Bartlett’s test, p < 0.05 [40]; the Hospital Survey on Patient Safety Culture in Croatia (HSOPSC-HRK) KMO = 0.887, Bartlett’s test, p < 0.001 [41], and the Hospital Survey on Patient Safety Culture in Dutch (HSOPS-NL) KMO = 0.9, Bartlett’s test, p < 0.001 [42]. The KMO and Bartlett’s test results of other studies in the literature showed compatibility with the results of this study.

The rate of the total variance in the measured variable explained by the factors of a scale, which is expected to be above 0.30 in multifactor constructs, indicates how well the related construct is measured, and this rate is expected to be high. The rates of the total variance explained by factors in various studies are as follows: HSOPSC-AR 61.44% [39]; HSOPS-TR 62.1% [21]; HSPSC-CN 60% [40]; HSOPSC-HRK 59% [41], and HSOPS-NL 57.1% [42]. These results were similar to the results obtained in this study. A high variance explanation rate shows how well the relevant construct is measured [28]. Hence, it can be stated that the total variance explained by the factors of the scale that was developed in this study was acceptable for the social sciences.

The factor load value is a coefficient explaining the relationship between items and factors, and it is expected to be high [28]. The existence of a cluster of items with high levels of correlation with a factor means that those items together measure a factor [28]. Some factor load value ranges in various cultural adaptations of scales were reported as follows: the Safety Attitudes Questionnaire (SAQ) between 0.40 and 0.81 [43]; the Safety Attitudes Questionnaire in Turkish (SAQ-TR) between 0.29 and 0.78 [20]; the Safety Attitudes Questionnaire in Danish (SAQ-DK) between 0.28 and 0.88 [44]; the Safety Attitudes Questionnaire German language version in Swiss (SAQ-CH) between 0.345 and 0.862 [45]; HSOPSC-AR between 0.43 and 0.88 [39]; HSOPS-TR between 0.36 and 0.87 [21]; HSPSC-CN between 0.40 and 0.70 [40]; HSOPSC-HRK between 0.365 and 0.908 [41]; HSOPS-NL between 0.36 and 0.88 [42], and the Hospital Survey on Patient Safety Culture (HSPSC-MX) between 0.324 and 0.970 [46]. All factor load values that were determined in this study were >0.30, and they were similar to the factor load values in previous scale development studies in the relevant literature.

The goodness-of-fit value refers to how well the parameter estimates of the results of CFA (i.e. factor loads, factor correlations, error covariances) reproduce the relationships observed in the sample’s data [37]. Among the model fit indices of this study, the chi-squared test statistic (χ^2^), χ^2^/df, comparative fit index (CFI), normed fit index (NFI), and incremental fit index (IFI) showed an excellent fit, while the root mean square error of approximation (RMSEA) and standardized root mean square residual (SRMR) values showed a good fit. In general, the goodness-of-fit indices of the scale in this study showed a good fit. In previous studies in which scales have been tested, the goodness-of-fit indices of SAQ [43], SAQ-TR [20], the Safety Attitudes Questionnaire in Chinese (SAQ-CN) [47], and the Safety Attitudes Questionnaire in Norwegian (SAQ-NO) [48] have shown satisfactory fit results. HSOPSC-AR [39], HSPSC-MX [46], the Safety Attitudes Questionnaire in Swedish (SAQ-SE) [49], SAQ-DK [44], and SAQ-CH [45] showed good fit results. Finally, goodness-of-fit values of the Patient Safety Climate in Healthcare Organizations (PSCHO) scale was found to be good [50].

The Cronbach’s alpha coefficient is used for the assessment of internal consistency. In the relevant literature, it was stated that Cronbach’s alpha coefficients in the range of 0.00–0.39 indicate that the examined scale is unreliable, those in the range of 0.40–0.59 indicate low reliability, those in the range of 0.60–0.79 indicate moderate reliability, and those in the range of 0.80–1.00 indicate high reliability [38]. In general, when this coefficient is 0.70 or higher, the results are considered reliable. As subscales, OLDC, MSL, and TW were determined as highly reliable, and WE, RPSE, NPWH, and RE were determined as quite reliable. However, it is also known that it is possible to obtain low Cronbach’s alpha coefficients when the number of items is low [51]. In previous studies, Cronbach’s alpha values of scales about a patient safety culture have been reported as HSOPSC-AR 0.87 [39]; HSOPS-TR 0.88 [21]; HSPSC-CN 0.84 [40]; HSOPSC-HRK 0.88 [41]; HSPSC-MX 0.71 [46]; SAQ-CN 0.945 [47]; SAQ-TR 0.89 [20]; and SAQ-DK 0.89 [44].

The aim of this study was to develop the Patient Safety Culture Scale for Hospitals (PSCSH) and test its validity and reliability. As a result of the psychometric properties of PSCSH, the scale was determined to be a valid and reliable measurement instrument for healthcare professionals. It is believed that the scale will be useful in determining the current patient safety culture at an institution and following up on its development, especially in healthcare institutions that will start initial patient safety practices.

## Figures and Tables

**Figure:**
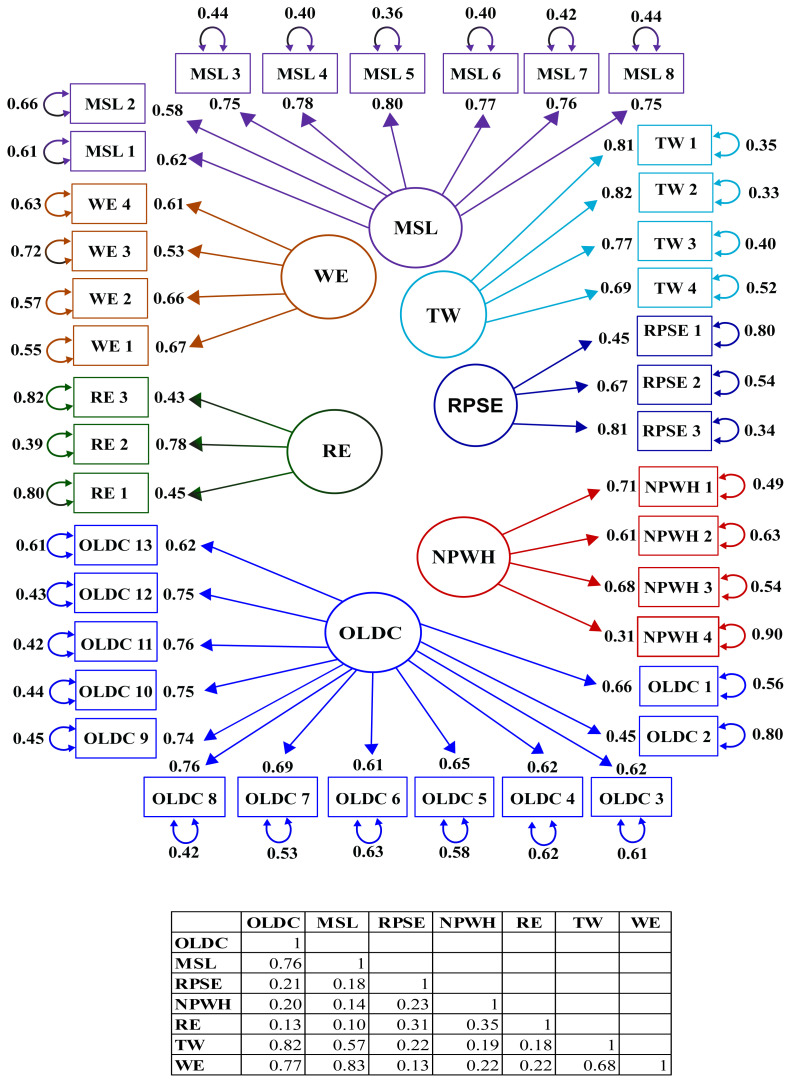
Standardized path coefficients.

**Table 1:** Factor loadings of the items.

Item number		Factor load
**1- Organizational Learning, Development, and Communication (OLDC)**		
I27	There is effective communication between this clinic and other clinics.	0.674
I28	Employees in this clinic trust each other while doing their jobs.	0.615
I33	Employees in this clinic work in compliance with patient safety processes.	0.583
I30	There is a continuous effort in this clinic to increase patient safety.	0.542
I35	Employees in this clinic share what they have learned with each other.	0.528
I32	In this clinic, the same medical errors are not allowed to appear again.	0.489
I26	In this clinic, all critical data of the patient are transferred along with the patient.	0.479
I41	There is no lack of communication among the employees in this clinic.	0.465
I19	In this clinic, an employee who makes an error is supported by colleagues.	0.451
I23	In this clinic, lessons are learned from errors.	0.373
I10	This clinic works in cooperation with other clinics.	0.370
I21	In this clinic, all critical data of the patients are kept in the information system.	0.362
I20	In this clinic, medical errors are discussed to eliminate the causes of errors.	0.325
**2- Management Support and Leadership (MSL)**		
I44	Hospital management considers it important to learn lessons from errors.	−0.780
I43	Hospital management deals with patient safety even though there are no negative incidents.	−0.732
I37	Hospital management investigates how to prevent medical errors.	−0.730
I39	Patient safety is among the top priorities of the hospital management.	−0.712
I31	Hospital management listens to employee’s recommendations to increase patient safety.	−0.485
I38	In this clinic, employees are informed about improvements made in patient safety.	−0.481
I6	Hospital management allocates sufficient resources for improving patient safety.	−0.451
I25	Hospital management uses an information management system to prevent medical errors.	−0.402
**3- Reporting Patient Safety Events (RPSE)**		
I42	In this clinic, if the medical error has not harmed the patient, it is not reported.	0.761
I24	In this clinic, if the medical error has not reached the patient ( near-miss), it is not reported.	0.744
I11	In this clinic, all medical errors are not reported.	0.509
**4- The Number of Personnel and Working Hours (NPWH)**		
I13	In this clinic, employees work beyond normal working hours.	0.739
I2	In this clinic, employees work more shifts than necessary.	0.651
I8	In this clinic, the number of personnel is insufficient.	0.476
I22	In this clinic, many temporary employees are employed.	0.311
**5- Response to Error (RE)**		
I14	In this clinic, an employee who makes a medical error thinks s/he is blamed.	0.423
I36	In this clinic, an employee who makes a medical error feels professionally incompetent.	0.380
I3	In this clinic, an employee who makes a medical error feels afraid of being punished.	0.342
**6- Teamwork (TW)**		
I1	In this clinic, employees cooperate while working.	−0.822
I4	In this clinic, employees help each other while working.	−0.794
I5	In this clinic, all employees at every level can warn others in terms of adversities in ensuring patient safety.	−0.483
I18	In this clinic, everyone treats others respectfully.	−0.386
**7- Working Environment (WE)**		
I9	In this clinic, sufficient training is provided on patient safety.	0.650
I7	In this clinic, care is taken to keep the working environment safe.	0.490
I12	In this clinic, there is always a sufficient amount of medical consumables.	0.415
I17	The physical condition in this clinic is suitable for working.	0.398

**Table 2:** Subsscales values of eigenvalue, variance explained, and Cronbach’s alpha.

Subsscales	Eigenvalue	Variance explained	Cronbach’s alpha
Organizational Learning, Development, and Communication (OLDC)	11.999	28.818	0.91
Management Support and Leadership (MSL)	3.347	6.395	0.90
Reporting Patient Safety Events (RPSE)	2.678	5.987	0.66
The Number of Personnel and Working Hours (NPWH)	1.551	2.495	0.66
Response to Error (RE)	1.508	2.999	0.66
Teamwork (TW)	1.126	1.856	0.85
Working Environment (WE)	1.099	1.373	0.71

**Table 3:** According to items confirmatory factor analysis t values.

New item number	Old item number	t value
OLDC 1	I10	17.32*
OLDC 2	I19	10.75*
OLDC 3	I20	15.98*
OLDC 4	I21	15.71*
OLDC 5	I23	16.75*
OLDC 6	I26	15.61*
OLDC 7	I27	18.12*
OLDC 8	I28	20.76*
OLDC 9	I30	19.97*
OLDC 10	I32	20.44*
OLDC 11	I33	20.81*
OLDC 12	I35	20.51*
OLDC 13	I41	15.90*
MSL 1	I6	15.81*
MSL 2	I25	14.55*
MSL 3	I31	20.31*
MSL 4	I37	21.36*
MSL 5	I38	22.40*
MSL 6	I39	21.19*
MSL 7	I43	20.79*
MSL 8	I44	20.24*
RPSE 1	I11	9.65*
RPSE 2	I24	13.82*
RPSE 3	I42	15.92*
NPWH 1	I2	15.41*
NPWH 2	I8	13.30*
NPWH 3	I13	14.72*
NPWH 4	I22	6.45*
RE 1	I3	8.58*
RE 2	I14	12.12*
RE 3	I36	8.22*
TW 1	I1	22.19*
TW 2	I4	22.74*
TW 3	I5	20.74*
TW4	I18	17.90*
WE 1	I7	16.51*
WE 2	I9	16.1*
WE 3	I12	12.44*
WE 4	I17	14.71*

* p < 0.05

**Table 4:** Model Fit Index.

Index	Acceptable level	Research finding	Conclusion
aχ^2^ p value	*χ*^2^ Value table	<0 .001	Perfect fit
*^a^χ^2^ / bdf	1.0 < *χ*^2^/df < 5.0	3.04	Perfect fit
**^c^RMSEA	0.05 ≤ RMSEA ≤ 0.08	0.06	Good fit
**^d^CFI	> 0.95	0.97	Perfect fit
**^e^NFI	0.90 ≤ NFI < 0.95	0.95	Perfect fit
***^f^IFI	> 0.95	0.97	Perfect fit
**^g^SRMR	<0.05	0.06	Good fit

* (Bollen, 1989; Mueller, 1996; Munro, 2005);

** (Schumacker & Lomax, 2016);

*** (Hoyle, 2012).

a χ^2^ chi-square test statistic;

b df, degrees of freedom;

c RMSEA, root mean square error of approximation;

d CFI, comparative fit index;

e NFI, normed fit index;

F IFI, incremental fit index;

g SRMR, standardized root mean square residual.
